# New Horizons in Plant Cell Signaling

**DOI:** 10.3390/ijms23105826

**Published:** 2022-05-23

**Authors:** Aloysius Wong, Christoph Gehring

**Affiliations:** 1 Department of Biology, College of Science and Technology, Wenzhou-Kean University, 88 Daxue Road, Wenzhou 325060, China; 2 Zhejiang Bioinformatics International Science and Technology Cooperation Center, Wenzhou 325060, China; 3 Wenzhou Municipal Key Lab for Applied Biomedical and Biopharmaceutical Informatics, Wenzhou 325060, China; 4 Department of Chemistry, Biology & Biotechnology, University of Perugia, Borgo XX Giugno, 74, 06121 Perugia, Italy

## Editorial on the Special Issue: New Horizons in Plant Cell Signaling

Responding to environmental stimuli with appropriate molecular mechanisms is essential to all life forms and particularly so in sessile organisms such as plants. To this end, plants have evolved both rapid early mechanisms such as the activation of channels and kinases directly or indirectly through protein sensors [1,2,3,4,5,6], as well as the slower systemic adaptive responses that include changes in their transcriptomes and proteomes [7,8,9,10,11,12]. To enable these processes and concomitantly tune their responses to the environment, complex cellular-signaling mechanisms have evolved, some of which have no homologues in animals [13,14,15,16,17,18,19,20,21]. This Special Issue aims to broaden the current understanding of plant cell signaling, specifically highlighting recent and exciting discoveries such as the identification of novel signaling molecules and mechanisms that participate across all stages of plant growth and development, and in cellular and biological processes triggered by abiotic and biotic stresses.

One such signaling molecule is the cyclic mononucleotide phosphodiesterases (PDEs), which, together with nucleotide cyclases, regulate the cellular concentrations of the second messengers, cyclic nucleotide monophosphates (cNMPs), cAMP and cGMP. While well-defined in bacteria, yeast and animals, the components of cNMP signaling pathways in plants are still poorly characterized [22,23,24,25,26]. The use of manually curated amino acid motifs based only on the catalytic centers of the corresponding enzymes in organisms across species have enabled the identification and concomitantly also the characterization of several novel adenylate cyclases (ACs) and guanylate cyclases (GCs) [27,28]. The latter is reviewed by Turek and Irving 2021 [29] within the context of moonlighting roles in modulating signal cascades. The authors discussed how the GC activities that moonlight within complex plant proteins, such as the receptor-like kinases and lipid kinases, can potentiate localized cGMP-enriched niches surrounding their primary domains and interactomes [30,31,32]. Such effects include the downregulation of kinase activity, the modulation of other components or complexes in the signaling pathway and the triggering of degradation cascades leading to signal termination [25,33]. The authors proposed that these moonlighting GCs which generate cGMP-enriched nanodomains in complex proteins form a new paradigm in homeostatic responses that enable a highly precise, spatially differentiated and stimulus-specific cellular signaling in plants [21,29,34,35]. 

While the generating enzymes of cyclic mononucleotides are being increasingly characterized, the degrading enzymes, the phosphodiesterases (PDEs), have remained elusive in vascular plants until recently. Previously, a novel protein harboring both AC and PDE activities known as CAPE (COMBINED AC with PDE) that may be involved in spermatogenesis and sperm motility, has been identified in the liverwort *Marchantia polymorpha* [36,37]. Adopting a motif-based approach similar to the one mentioned above, Kwiatkowski et al., 2021 [38] reported that a monocot *Brachypodium distachyon* (BdPDE1) can hydrolyze cNMPs to 5′NMPs but with a preference for cAMP over cGMP. Much like the PDE activities of other systems [39], BdPDE1 activity was significantly enhanced by Ca^2+^-calmodulin. Mutagenesis studies also identified and revealed the importance of several key residues in the catalytic center. Based on the biochemical, mutagenesis and structural analyses, as well as cross-species sequence analyses, the authors have deduced an amino acid consensus sequence that can be applied in eukaryotes and prokaryotes. Identifying functional PDEs in monocots is a significant step towards crop biotechnology, e.g., enabling the design of specific inhibitors with a view to developing improved crops. Previously, a tandem motif and structural approach has identified a functional PDE moonlighting at the C-terminal of a potassium transporter from the model plant *Arabidopsis thaliana* (AtKUP5) which also harbors a functional AC at the N-terminal [40,41]. The discovery of such dual moonlighting AC-PDE architecture in complex proteins raises the exciting possibility of providing an intricate and dynamic localized spatiotemporal fine-tuning mechanism for signal intensity [30,31,42,43]. Recent discoveries of PDEs in both monocots and dicots suggest that this PDE architecture may well be a common feature in higher plants and that this motif-based strategy can be used to identify novel PDEs in model and crop plants. 

The non-cyclic form of cAMP, adenosine monophosphate (AMP), is present in all life forms and is central for energy metabolism achieved through the enzyme 5’ adenosine monophosphate-activated protein kinase (AMPK). As an energy sensor, AMPK is activated by decreasing ATP or increasing AMP and ADP [44,45,46]. AMP is a direct agonist for AMPK and its activation by AMP has been observed through allosteric regulation in various organisms [47]. In this regard, Clark et al., 2020 [48] reviewed the enzymes that generate AMP through the hydrolysis of ATP in plants. They are known as apyrases (APYs). The authors provided a historical account of plant APYs with a particular focus on the progress that has been made in their biochemistry, structures, and functions, reported between 2015 and 2020. Among the recent studies described were reports on how APY expression is linked to growth through extracellular ATP (eATP) treatments and how APY exerts a protective role in plant biotic and abiotic stress responses through the induction of gene expression changes while also appearing to crosstalk with hormones such as ethylene and auxin [49,50,51,52,53]. The authors noted that the initial discovery of plant eATP receptor in *Arabidopsis thaliana*, AtAPY1 [54], has encouraged research on APYs and despite several contradictory reports [55,56], recent data supported the ability of AtAPY1 to bind and hydrolyze ATP [57]. Additionally, new data on APY functions in multiple plant species such as peas, Brassica, and poplar assign the important role of APYs in plant defense responses [58,59,60]. These, and other major recent advances including the availability of new crystal structures, offer insights on the NTP-binding domain [57], the ability of APYs to hydrolyze ATP in the ECM and nuclei [60], and the identification of APY-binding partners [61]. The findings are summarized in a table. The authors also reviewed the prospect of using APY-specific inhibitors and their value in future research that focuses on how APY regulates cellular activities. The state of the current field is also framed in the form of outstanding major questions that serve to guide research in the field of plant APYs. 

Salicylic acid (SA), a phenolic plant hormone found in many plant species, functions as a critical signaling molecule in local and systemic disease resistance pathways [62]. Responding to a broad spectrum of pathogens and abiotic stresses, SA also crosstalks with many signaling pathways ranging from reactive oxygen species, lipids, and circadian clock to other hormones such as jasmonic acid and ethylene [63,64,65,66,67]. Plant defense signaling by SA is initiated when it binds to target proteins such as the nonexpresser of pathogenesis-related protein 1 (NPR1) which is a well-established transcriptional regulator of SA signaling [68]. Pokotylo et al., 2020 [69] proposed that SA could bind to many other proteins, some of which are enzymes involved in primary metabolism such as the glyceraldehyde 3-phosphate dehydrogenase (GAPDH) which was previously shown to bind SA in both humans and plants [70,71,72]. The authors reported that GAPDH activity was inhibited by SA and showed in surface plasmon resonance studies that SA binds to the A1 isomer of a chloroplastic glyceraldehyde 3-phosphate dehydrogenase (GAPA1) from *Arabidopsis thaliana* with a *K_D_* of 16.7 nM. The authors further revealed two putative binding pockets through molecular docking and molecular dynamics simulations on the apoprotein and protein–ligand complex, but only one pocket around Asn35 could bind SA when simulated in an aqueous environment. This pocket is also the binding site of the NADP^+^ cofactor and the binding of SA to GAPA1 inhibited NADH in a dose–response manner. Mutagenesis simulations assigned key roles to Asn35 and Arg81 for binding of SA to GAPA1 which the authors then used as a guide for the subsequent in vitro biochemical validations. Mutations of these two residues markedly reduced the ability of GAPA1 to bind SA. Taken together, this research offers novel insights into how SA controls energy fluxes in stressed plants while also providing a new dimension to the current paradigm of SA signaling through its interaction with proteins which have not normally been associated with SA [73,74]. 

Another signaling molecule, Ca^2+^, has long been known to have a significant role in many cellular activities in both plant and animal systems signaling for cell division, cell movement, cell death, fertilization, and metabolism [75,76]. Ca^2+^ ions in the cytosol of a resting cell are maintained at very low levels typically about 10,000 times lower than that at the extracellular space, or in the vacuole and the lumen of intracellular stores. As such, cytosolic Ca^2+^ must be tightly regulated by the presence of various Ca^2+^ ion pumps, exchangers, and channels. Ca^2+^ entry can be activated by membrane hyperpolarization among other possible mechanisms, but unlike the more common depolarization activation, membrane hyperpolarization would mean that hyperpolarization-activated Ca^2+^ channels (HACCs) are normally kept open in the resting state of the membrane [77,78,79]. If not regulated, HACCs, which are also permeable to Ba^2+^ and Mg^2+^, would leak Ca^2+^ into cells. Lemtiri-Chlieh et al., 2020 [80] showed that Mg-ATP, but not ATP on its own, significantly reduces HACC activity especially at −200 mV or lower in *Vicia faba* guard cells and this effect is specifically due to Mg^2+^. It led the authors to conclude that Mg^2+^ prevents the continuous leakage of Ca^2+^ into the cells through the inactivation of HACCs and that the opening of these channels would require high negative voltages or displacement of Mg^2+^. Through structural analysis of other Mg^2+^-sensitive K^+^ channels, the authors proposed that the particular HACCs responsible for this result in the guard cell are the cyclic nucleotide-gated channel (CNGCs) which harbor a conserved Mg^2+^-binding motif within their pores [81,82,83]. In the guard cell, HACCs can be activated by the sesquiterpene hormone ABA and H_2_O_2_ [84,85]. Therefore, this study establishes an important role for Mg^2+^ in Ca^2+^ signaling and in plant physiological processes including ABA-dependent responses which might bridge existing gaps concerning Ca^2+^ homeostasis in the current literature [75]. 

Being immobile, plants are constantly exposed to biotic and abiotic stresses and must also respond to a changing external environment. As the boundary between the plant and the external environment, the plant epidermis is not just a barrier that protects the plant against pathogens, but it also regulates exchange of water, nutrients, and gases crucial for growth and development processes [86,87]. The mechanisms involved in transcriptional regulation of epidermal cells in processes such as shoot growth, lipid metabolism and cuticle synthesis, as well as defense responses, are largely unknown [88]. Wang et al., 2020 [89] dissected the promoter of *Triticum aestivum* lipid transfer protein 1 (TaLTP1) by generating multiple deletion constructs and studying their activities in transgenic *Arabidopsis thaliana* and *Brachypodium distachyon*. Through histochemical GUS and quantitative fluorometric analyses, the −400TaLTP1::uidA construct was able to confer full activity at the vegetative stage. A separate construct, −343TaLTP1::uidA, caused a loss of quantitative GUS activity by about 90% in transgenic plant leaves which is associated with pavement cells but not trichomes. Another construct, −297TaLTP1::uidA, resulted in a complete loss of GUS activity in true leaves but the activity was not altered in cotyledon until the promoter region was shortened to position −247 bp. Through mutagenesis studies, the authors also identified the specific *cis*-elements which they named as GC(N_4_)GGCC at positions −380 bp to −372 bp, that are responsible for pavement-cell-specific expression. Using electrophoretic mobility shift and transgenic reporter assays, the authors found a CCaacAt motif at −303 bp that regulates trichome-specific expression while a conserved CcATC motif at −268 bp was found to be involved in regulating pavement-cell-specific expression. In summary, this study identified the specific promoter regions of TaLTP1 responsible for tissue-specific expression as well as the *cis* elements responsible for epidermal-cell-specific expression in shoots, thus contributing to the broader understanding of gene transcription regulation in plant epidermis of aerial tissues [90,91].

Another exciting development related to the regulation of gene expression are the microRNAs. Present in both animals and plants, these single-stranded non-coding RNAs are involved in silencing and post-transcriptional regulation of RNAs [92,93]. Han and Zhou (2022) [94] reviewed the recent progress of one such group of RNAs, the microRNA171 (miR171) in plants. The authors described that miR171 is ancient and conserved in land plants and exerts its regulatory effects by repressing the HAIRYMERISTEM (HAM) gene family [95,96,97,98]. In *Arabidopsis thaliana*, miR171 acts as a mobile short-range signal that initiates the epidermal layer of shoot meristems and affects the patterning of apical–basal polarity of gene expression and stem cell dynamics [99]. Besides providing a brief account of miRNA171 and its targets, the authors also discussed its function as a regulatory hub in diverse plant developmental processes, as well as their expression patterns and regulations in response to abiotic stresses such as light. The authors noted that miR171 family members are conserved and have lineage-specific functions in land plants. Thus, they proposed, among other research directions, to focus on how miR171 connects environmental factors to plant development not just in seed plants but also in in non-seed vascular plants such as the fern *Ceratopteris richardii.* This may well provide a better account of its evolution in land plants and could potentially make miR171 a target for crop improvement and protection [100,101,102]. 

Contributions in this Special Issue have revealed novel signaling molecules, signaling mechanisms, and regulations that broaden our view on how plants signal for growth and development, and their responses to environmental stresses. The body of research presented in this series will hopefully inspire hypothesis generation as well as encourage and guide experiments that will collectively advance our understanding in the rapidly evolving field of plant cell signaling.
